# Statistical Experimental Design Optimization of Microbial Proteases Production under Co-Culture Conditions for Chitin Recovery from Speckled Shrimp *Metapenaeus monoceros* By-Product

**DOI:** 10.1155/2020/3707804

**Published:** 2020-01-22

**Authors:** Fadoua Jabeur, Sondes Mechri, Mouna Kriaa, Ines Gharbi, Nejla Bejaoui, Saloua Sadok, Bassem Jaouadi

**Affiliations:** ^1^Laboratory of Microbial Biotechnology and Engineering Enzymes (LMBEE), Centre of Biotechnology of Sfax (CBS), University of Sfax, Road of Sidi Mansour Km 6, P.O. Box 1177, Sfax 3018, Tunisia; ^2^Laboratory of Microorganisms and Biomolecules (LMB), Centre of Biotechnology of Sfax (CBS), University of Sfax, Road of Sidi Mansour Km 6, P.O. Box 1177, Sfax 3018, Tunisia; ^3^Institut National Agronomique de Tunis (INAT), Université de Carthage, 43 Avenue Charles Nicolle, 1082 Tunis Maharajène, Tunisia; ^4^Laboratory of Blue Biotechnology & Aquatic Bioproducts (B3Aqua), Institut National des Sciences et Technologies de la Mer (INSTM), Annexe La Goulette Port de Pêche, La Goulette 2060, Tunisia

## Abstract

This study was designed with the aim to produce microbial proteases in presence of speckled shrimp by-product. For this reason, three strains belonging to *Bacillus* genus, namely, *Aeribacillus pallidus* VP3, *Lysinibacillus fusiformis* C250R, and *Anoxybacillus kamchatkensis* M1V were studied under co-culture procedure. A Taguchi L27 experimental design was applied to optimize the co-culture parameters. The experimental design was built with 9 factors (by-product powder concentration, the pH of the medium, the temperature, the sucrose concentration, the agitation speed, the inoculum sizes of VP3, M1V, and C250R strains, and the culture volume) at three different levels. The obtained results showed that a total protease activity of 8,182 U/mL could be achieved after 24 h of incubation in presence of 20 g/L shrimp by-product and 10 g/L sucrose, at an initial pH of 7, a 40°C temperature and absorbance, at 600 nm, of inoculum sizes of 0.1, 0.3, and 0.1 for VP3, M1V, and C250R strains, respectively. The agitation was set at 200 rpm, and the final volume was 25 mL. Taguchi's design allowed the identification of temperature, the inoculum size for strain VP3, the inoculum size for strain M1V, and the final culture volume as the most influencing variables. A Box–Behnken design with 27 experiments was carried out for the optimization of these four selected factors. Following such design, the highest protease production reached was 11,300 U/mL. This yield was obtained in a final culture volume of 15 mL containing 20 g/L shrimp by-product powder and 10 g/L sucrose and inoculated with VP3, C250R, and M1V strains at 0.05, 0.1, and 0.2, respectively. The flasks were incubated at 45°C for 24 h with shaking at 200 rpm. The efficiency of chitin extraction by co-cultivation was investigated under the latter conditions. The chitin yield from shells by-product was 16.7%. Fourier-Transform Infrared (FTIR) analysis of the obtained chitin displayed characteristic profiles similar to that of the commercial *α*-chitin.

## 1. Introduction

Environmental pollution has lately become a great concern. Indeed, many industrial wastes have contaminated the natural environment, therefore seriously threatening human lives. The released pollutants remain a serious problem in both industrialized and developing countries [1, 2]. The control of water pollution and the recovery of industrial residues are important objectives in most countries. To achieve this, technologies for waste treatment and valorization are highly searched. In particular, the processing and handling of marine by-products are being discussed. In fact, marine by-products are nonedible parts of fish, crustaceans, or cephalopods including skin, heads, bones, internal skeletons, scales, tails, shells, and viscera, being thrown during industrial processing [3]. In recent years, biotechnology has made possible to exploit marine biowastes for the recovery of natural macromolecules such as oils, antibiotics, enzymes, bioactive peptides, as well as gelatin, chitin, and its derivatives, which are with great interest at the pharmaceutical, biomedical, agricultural, and environmental sectors [4]. For particular interest, chitin, which is a polymer formed from a repeating unit of *N*-acetyl-d-glucosamine, is the second most abundant organic compound after cellulose. The main sources of raw materials for chitin production are crustacean cuticles, mainly crabs and shrimps [5, 6]. However, it was discovered after isolation from superior fungi in 1811 by Braconnot as cited in other studies [7]. Chitin occurs naturally in crystalline form or in the form of chitino-protein complexes. It is a rigid organized fibers network. This rigidity confers insolubility to chitin in most solvents. Different arrangements of chitin chains are possible. They have helical forms, and they are all directed along the same axis giving rise to three chitin allomorphs *α*, *β*, and *γ* [8]. The typical chitin extraction methods require the use of chemicals such as hydrochloric acid (HCl), acetic acid, and caustic soda (NaOH) which are pollutants of the aquatic ecosystem and endanger the fauna and the flora [9]. In order to overcome the adverse effects of the chemical treatment, biologists have resorted to the use of proteolytic enzymes for the deproteinization of shrimp waste [10]. Enzymatic methods include the use of several enzymes such as alcalase, trypsin, papain, and pepsin. However, the elevated cost and the low extraction efficiency are the main disadvantages of the process [11]. Recently, the application of proteases and lactic acid-producing bacteria, to extract chitin from crustacean by-products, has gained an increasing interest. Actually, this method has the advantage of being relatively simple and less expensive and using a chemical-free process [12, 13]. Hence, in comparison with chemical and enzymatic methods, it has been found that microbial fermentation methods are the most effective for chitin extraction, especially, in terms of final product quality and environment safety [14]. Liu et al. have applied successive co-fermentation of a protease-producing bacterium*, Bacillus licheniformis* 21886, and an acid-producing bacterium, *Gluconobacter oxydans* DSM-2003, for the extraction of chitin from shrimp by-products. These authors revealed that the biological methods were able to preserve the quality of chitin and its derivatives resulting in a liquor fraction rich in proteins, minerals, and amino acids, as well as a solid chitin fraction. Furthermore, Tao et al. applied the co-cultivation of indigenous bacterial consortium and exogenous *Bacillus subtilis* in order to investigate the biodegradation of crude oil [15]. Thus, the results of these works showed a promising potential of co-cultivation method when proposed for bioremediation applications.

In recent years, there has been an increasing interest in the use of statistical tools, such as the methodology of experiments, in the analysis of scientific research results in several fields. The statistical experiment plans make it possible to obtain as much information as possible while minimizing the number of experiments to be performed. In this context, the use of experimental design in the field of marine biotechnology is increasing over the years [10] Therefore, the objectives of this study were the application of Taguchi and Box–Behnken designs to optimize the production of proteases by the co-cultivation of three proteolytic bacteria, *Aeribacillus pallidus* VP3, *Lysinibacillus fusiformis* C250R, and *Anoxybacillus kamchatkensis* M1V grown on medium containing shrimp *Metapenaeus monoceros* by-product, for chitin recovery.

## 2. Experimental Section

### 2.1. Substrates, Reagents, and Shrimp Shell By-Products

All substrates and chemicals were reagent grade unless specified otherwise. Commercial chitin was purchased from P-Biomedical, France. Casein for the assay of protease activity was supplied by Merck (Darmstadt, Germany).

Fresh shells from speckled shrimp *Metapenaeus monoceros* were obtained from a fish market in Sfax at the south of Tunisia. Shrimps are generally processed, generating large part of co-products represented principally by shells [16]. In this work, shells were washed thoroughly with tap water, cooked at 90°C for 20 min (1 : 2 ratio (w/v)) to inactivate endogenous enzymes, then dried at room temperature, and milled with a Stainless Steel Cross Beater Mill Model Sk1 1.1 kW 1 to obtain a fine and homogeneous powder with particle sizes <1.23 mm [5]. After drying, the powder was stored at room temperature until use.

### 2.2. Microorganisms

Three bacterial strains, namely, *Aeribacillus pallidus* VP3, *Lysinibacillus fusiformis* C250R, and *Anoxybacillus kamchatkensis* M1V, were used in this study. *Aeribacillus pallidus* VP3 and *Lysinibacillus fusiformis* C250R were isolated from the production water of the oilfields Thyna Petroleum Services (TPS), Sfax, Tunisia [17, 18]. *Anoxybacillus kamchatkensis* M1V was isolated from the Hammam Righa geothermal waters in Algeria [19]. All strains have been previously reported as extracellular proteases producers. However, they did not show any chitinase activity [19–21].

### 2.3. Protein, Lipid, and Sugar Analyses

Shrimp by-product was characterized by the determination of total proteins, total sugars, and total lipids. Protein was measured as total nitrogen content using the Kjeldahl method [5]. Total lipids content was determined using the Folch method [22]. Total sugar content was determined using the Dubois method [23].

### 2.4. Growth Conditions of Protease-Producing Bacterial Strains

To perform the precultures, one colony of each strain agar culture was added to 100 mL of Luria–Bertani (LB) medium composed of peptone 10 g/L, yeast extract 5 g/L, and NaCl 5 g/L at pH 7.4 in 500 mL Erlenmeyer flasks and incubated on a shaker incubator at 45°C for VP3 and M1V strains and at 37°C for strain C250R during 12 h. Liquid mixed cultures were established by adding simultaneously precultures of VP3, C250R, and M1V strains to fermentative medium. To establish co-cultivation, a preliminary point experiment was performed in 500 mL Erlenmeyer flasks by an initial inoculum size (0.2) for each strain. In fact, VP3, C250R, and M1V precultures were transferred simultaneously to 100 mL of medium and incubated at 45°C at 200 rpm for 24 h. In this optimal condition, the maximum proteases production was 2,845 U/mL.

### 2.5. Assay of Proteolytic Activity

Before each assay, the cell debris was removed by centrifugation of the co-cultivation at 10,000 ×*g* for 30 min, and the supernatant was used for estimation of protease activity. Using casein as a substrate, the protease activity was routinely evaluated by the modified method described elsewhere [24]. Briefly, a suitably diluted enzyme (0.5 mL) was mixed with 100 mM glycine-NaOH buffer (0.5 mL) containing 10 g/L casein (0.5 mL) for 15 min at optimum temperature. The reaction was then stopped by the addition of 0.5 mL trichloroacetic acid (TCA) 20% (w/v). The mixture was left to rest for 15 min at room temperature (∼25°C) and then centrifuged at 10,000 ×*g* for 15 min to remove the precipitate. The acid soluble material was measured spectrophotometrically at 280 nm. One unit (U) of protease activity was defined as the amount of enzyme required to release 1 *μ*g tyrosine per minute under the experimental conditions used.

### 2.6. Optimization of Enzyme Production

#### 2.6.1. Influence of Shrimp By-Product and Sugar-Induced Concentration

For a higher production of protease activity, the best concentrations of shrimp by-product and sugar used as a carbon and nitrogen sources were chosen using the traditional method “one variable at a time,” for each of the three used strains. The experiments were carried out in 500 mL Erlenmeyer flasks containing 100 mL of liquid production medium. After sterilization, the flasks were inoculated and maintained under different conditions using various concentrations of shrimp by-product (From 10 to 40 g/L) and a single sugar concentration of 10 g/L.

#### 2.6.2. Selection of Significant Factors by Taguchi's Design

For screening of the most influencing factors, a Taguchi experimental plan with 27 experiments (9 factors at three-level) was performed using the protease activity production as the response of the model. The factors are the shrimp by-product powder concentration, the pH of the medium, the temperature, sucrose concentration, the agitation speed, the inoculum size for each used strain, and the culture volume.

#### 2.6.3. Response Surface Methodology Using Box–Behnken Design

The validity of the proteases production is confirmed by the application of optimal conditions (each factor at its optimal level). Subsequently to such optimal conditions, a Box–Behnken design with 27 experiments (4 factors at 3 levels) was applied to determine the optimal levels of the preselected factors. The absorbance at 600 nm of optimized inoculums sizes for VP3, M1V, and C250R strains is 0.1, 0.3, and 0.1, respectively.

#### 2.6.4. Chitin Recovery

After validation of RSM model, the proteases production was undertaken under the optimal conditions. At the end of the culture, the medium was centrifuged; the pellets were washed with distilled water and filtered to remove bacteria. The retentate rich in chitin was then dried for 48 h at 60°C. Chitin yield was calculated as chitin derived in reference to the original wet sample quantity of shells as detailed previously [25].

#### 2.6.5. FTIR

Infrared spectra of commercial and extracted chitins were determined using a Nicolet FTIR spectrometer equipped with a horizontal attenuated total reflection (ATR) accessory. The analysis of our samples was carried out at a scan range from 400 to 4,000 cm^−1^ in order to identify the characteristic functional groups [26]. This technique allowed the simultaneous collection of spectral data following the deproteinization of shrimp by-product in comparison with the profile of commercial chitin.

## 3. Results and Discussion

### 3.1. Chemical Composition of Shrimp By-Product Powder

The analysis of the biochemical composition of the shrimp by-products powder showed significant amounts of proteins (122.9 mg/g), lipids (40 mg/g), and sugars (18 mg/g). As the profitability of industrial fermentation processes is closely linked to the choice of the culture medium, it has been proposed to use shrimp by-product to prepare a powder for the formulation of an economically affordable culture media for the production of bacterial proteases for chitin recovery [27]. Indeed, during processing and packaging, nearly half of crustacean fresh mass is discarded as waste. In the absence of biotechnological recovery processes, this biowaste represents a heavy burden for the ecosystem as an additional source of anthropogenic organic matter.

### 3.2. Co-Culture of VP3, C250R, and M1V Strains on Solid LB Medium

**Figure 1:**
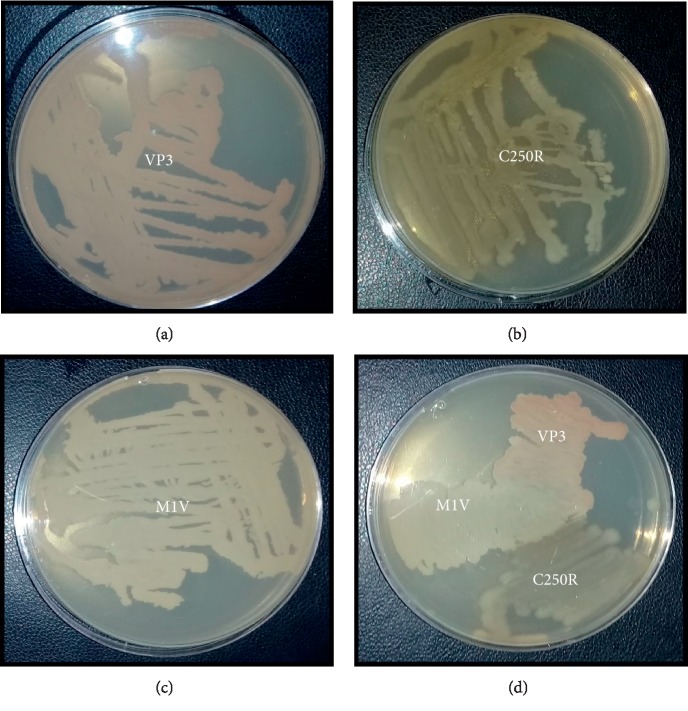
Co-cultivation of three strains on solid LB medium (a) *Aeribacillus pallidus* VP3, (b) *Lysinibacillus fusiformis* C250R, (c) *Anoxybacillus kamchatkensis* M1V, and (d) VP3, C250R, and M1V strains.

To determine whether the strains were able to grow as mixed cultures, several LB plates were inoculated.  Figure 1 shows that the strains are able to grow together without the appearance of any inhibition zone. When they were inoculated at a distance away from each other, the edges of the colonies of VP3, C250R, and M1V were able to grow through each other in the zone where the colonies would meet, but they would not strongly invade the space occupied by the other bacteria.

### 3.3. Production of Proteases in Economical Medium

The *Anoxybacillus kamchatkensis* M1V was found to produce high level of protease activity when grown in media containing only shrimp waste powder as presented in Table 1. A maximum protease activity of 2,000 U/mL was obtained with 25 g/L shrimp waste. The VP3 and C250R strains also produce large amounts of proteases activity in the order of 600 and 200 U/mL (Mechri et al. [13, 19], submitted for publication), respectively. *Bacillus licheniformis* RP1 was shown to produce proteases (1,400 U/mL) when grown in media containing shrimp wastes powder as carbon and nitrogen source, indicating that this bacteria could obtain its needs directly from shrimp by-product [28]. Also, Souissi et al. has used cuttlefish powder from *Sepia officinalis* by-products as a substrate for microbial growth and protease production by several bacteria: *Bacillus licheniformis*, *Bacillus subtilis*, *Pseudomonas aeruginosa*, *Bacillus cereus*, and *Vibrio parahaemolyticus* [29]. All microorganisms studied grew well and produced protease activity when grown in a medium containing only cuttlefish by-product powder with a production rate of 821; 392; 1,684; 2,771; and 2,487 U/mL, respectively.

Growth and proteases production were performed on medium containing only 25 g/L of shrimp by-product powder. The co-cultivations were carried out at 45°C with an initial absorbance at 600 nm of about 0.2 for each strain, in 500 mL Erlenmeyer flasks with a working volume of 50 mL. Regular samples were withdrawn, and bacterial growth and protease activities were determined. The optimum production of the protease activity was found to be 2,845 U/mL after 24 h, and it was coupled with the bacterial growth. In order to study the production of proteases by co-cultivation of the three strains VP3, C250R, and M1V on powder from white shrimp, the effect of various concentrations of this biowaste was investigated. The optimum protease activity production (5,000 U/mL) was obtained at 25 g/L with a significant yield of 80,000 IU/g. The effect of the addition of easily assimilable sugar on protease activity was also studied. The addition of sucrose and glucose enhanced the production of protease activity (from 5,000 to 5,300 U/mL), as well as the bacterial growth. Since sucrose is less expensive than glucose, we chose to continue working with sucrose as an inducing sugar for the production of proteases by co-cultivation of VP3, C250R, and M1V strains.

### 3.4. Optimization of Co-Culture Conditions for Protease Production

#### 3.4.1. Taguchi's Design Screening

Nine factors were selected to study their effect on the production of proteases and to determine their optimal levels by means of the Taguchi's L27 method (27 experiments and 9 factors with 3 levels). Information regarding the definition of the different factors and their studied levels are represented in Table 2. The experimental responses of the L27 Taguchi's design are presented in Table 3. Taguchi's design is a screening tool applied to identify, among a large number of potentially influencing factors, including those that appear to have a minor effect. Taguchi's method is utilized in many fields of engineering to improve product quality at the design stage. Its contribution consists essentially in defining the robustness of the products and the principles of design which lead to such robustness. The application of Taguchi's design in the field of biotechnology, especially in the fermentation processes, was proven to be extremely useful [30]. The analysis of the results of the Taguchi L27 plan (Table 3) showed that the production of proteases strictly depends on the co-cultivation conditions corresponding to the cumulative effect of the factors at a tested level. Thus, a remarkable variation of enzymatic activity was noted among the 27 experiments. This variation confirms that the production of proteolytic activity is closely related to the levels of studied factors. Such analysis showed also that the different studied parameters have significant effects on the production of proteolytic activity. Indeed, a temperature of 40°C and inoculum sizes of the VP3 and M1V strains of 0.1 and 0.3, respectively, with a culture volume of 25 mL are the most influencing factors on the protease activity production. Actually, these factors have a positive effect on protease production (8,182 U/mL) when present in the culture medium (Run 20). This is likely to be more justified by the absence of protease activity in run number 19 (Table 3).

The analysis of the Taguchi plan results (Table 4) confirmed that the production of proteases in a shrimp waste-based medium depends on several factors, mainly 4 factors, temperature, inoculum size for strains VP3, inoculum size for strain M1V, and volume of culture as their contribution totalized more than 97%. For all of these factors, the most important response is obtained when they are at their first level for the volume of culture and the size of the inoculum of VP3 and at the intermediate level (2) for the temperature with the exception of the size of the inoculum of M1V where the optimal level is 3. A remarkable difference is noted between the response at the second level and the response at the first level (L2 and L1) for the factors, temperature, pH of the medium, agitation, and the inoculum size for M1V strain, which are in the order of 1,973, 1,487.33, 1,177.67, and 1,162.56, respectively. This difference illustrated the existence of a variation in production between the different levels studied, which confirmed the effect of the level of such factors on the production of proteases for co-cultivation of VP3, M1V, and C250R strains.

#### 3.4.2. Validation of Optimal Conditions Selected by Taguchi's Design

The optimum protease production was validated by the application of the optimal conditions for the different factors, namely, shrimp biowaste at 20 g/L, sucrose at 10 g/L, initial medium pH at 9, with an absorbance at 600 nm of inoculums sizes 0.1, 0.3, and 0.1 for VP3, M1V, and C250R strains, respectively, an incubation at 40°C under 200 rpm agitation, and a working volume of 25 mL in Erlenmeyer flask of 500 mL. The medium was harvested after 24 h. The results showed that the optimization of production of proteases by the co-fermentation was very successful as the level of proteases production (10,922 U/mL) was so far higher than that obtained during the preliminary study (5,300 U/mL). The analysis of the results also showed that the experimental and the theoretical values are very close.

#### 3.4.3. Response Surface Methodology Using Box–Behnken Design

The optimization of the most influencing factors was carried out using a Box–Behnken design (27 experiments and 4 factors at 3 levels). The definition of the studied factors and their levels are illustrated in Table 5. The Box–Behnken planned experiments and their responses are given in Table 6.

#### 3.4.4. Statistical Analysis

**Figure 2:**
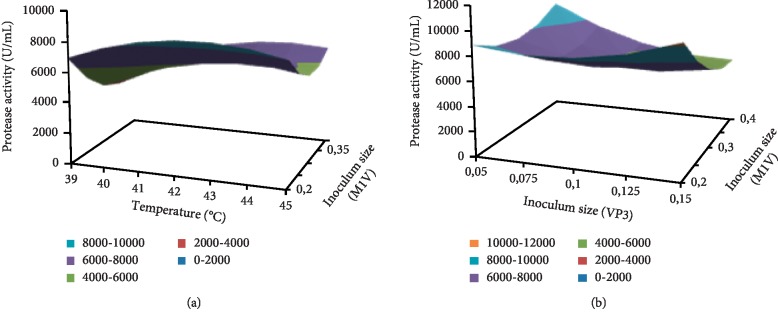
Response surface plot of proteolytic enzyme production showing the interactive effects of the inoculum sizes of strains M1V and VP3 (a); and inoculum size of strain M1V and temperature (b).

The results obtained following the application of the Box–Behnken design were mathematically modeled as a nonlinear regression using the SPSS program (Version 11.0.1.2001, LEAD Technology, Inc.,). This model takes into consideration the primary and the secondary effects of each factor as well as the second order interaction between the different factors (Figures 2(a) and 2(b)).

The established model was predicted by the following equation:

(1) $$\begin{array}{c} Y=-188459.48+10226.15\times X1\;101030.287698455\times X3\;233.85\times X4+127.03\times X1\times X1\;170.95\times X1\times X2+1555\;59\times X1\times X3+12.1\times X1\times X4+425711.90\times X2\times X2\;599867.36\times X2\times X3\;382.45\times X2\times X4+141817.26\times X3\times X3-7.91\times X4\times X4\;32.52\times X1\times X3\times X4+315.01\times X1\times X2\times X3\times X4, \end{array}$$

where *Y* is the protease activity (U/mL) and *X*1, *X*2, *X*3, and *X*4 are the temperature, the inoculum size of strain VP3, the inoculum size of strain M1V, and the final culture volume, respectively. The regression analysis showed an *F* value of 8.467 with a very low probability value (*P* < 0.001) indicating that the model is highly significant. The closeness of experimental and predicted protease activity was expressed by the regression coefficient of (*R*^2^ = **0.953**) which denotes that only 0.047% of the total variation could not be explained by the established model. The adjusted *R* square (predicted *R*^2^) of 0.816 explains the good agreement between the experimental and the predicted results. According to this model, the best protease activity could be achieved at 20 g/L shrimp by-product, 10 g/L sucrose, in a final volume of 15 mL with a pH of 9, and in the presence of initial inoculum sizes for strains VP3, C250R, and M1V equal to 0.05, 0.1, and 0.2, respectively, and with an incubation at 42°C under an agitation of 200 rpm.

#### 3.4.5. Validation of the Model Predicted by Box–Behnken's Design

The optimal conditions, determined in the previous study, were applied in two experiments while maintaining the conditions already established in the screening and optimization study by Taguchi and Box-Behnken designs. The protease activity, obtained under optimal conditions during the co-cultivation of VP3, M1V, and C250R strains, was much better than that obtained before optimization. Thus, the level of protease production was multiplied by a factor of 3.96 times (11,365 U/mL). The response surface plot is generally the graphical representation of the regression equation, from which the response (protease production) is plotted against two variables only, while other variables are fixed at the level 0 coded value. Figures 2(a) and 2(b) show the mutual interaction between the temperature and the inoculum size of M1V on one hand and between inoculum sizes of M1V and VP3 on the other hand, respectively. Thus, a relatively important protease production is recorded at the minimum levels of both factors for the two graphs. Consequently, the protease activity variation was observed with the simultaneous decrease or increase of these two factors. The contour map traits can reflect the strength of the interaction between the two factors: an oval contour reflects that the interaction between both factors is strong, whilst a circular contour indicates that the interaction between the two factors is weak [31].

### 3.5. Chitin Yield and Characterization by FTIR

**Figure 3:**
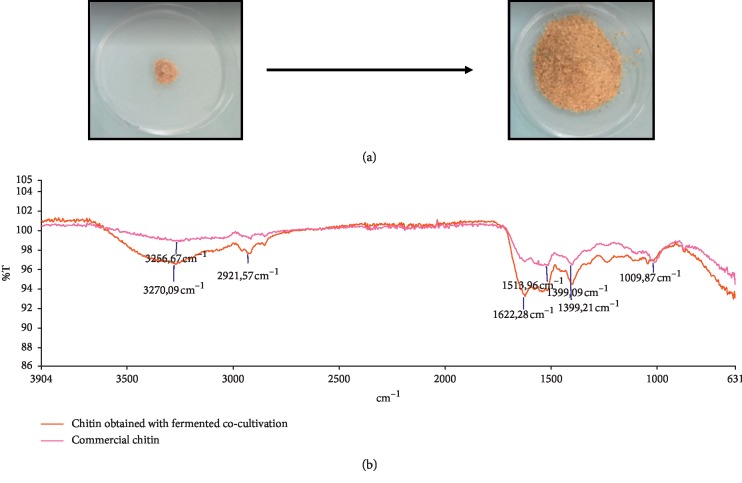
(a) (A) Shrimp by-product powder; (B) chitin obtained by co-fermentation. (b) FTIR spectra of chitin recovered from shrimp by-product treated with co-culture and commercial *α*-chitin.

The pellet of the co-cultivation is filtered to remove the bacteria and then dried to recover pure chitin (Figure 3(a)) with a yield estimated to 16.7%. Such percentage was comparable to that previously reported in other studies such as the chitin extracted (16.2%) from snow crab waste, *Chionoecetes opilio*, [32] and from prawn shell waste (22.4%) [33]. The structure of the chitin extracted from *Metapenaeus monoceros* by-product following the co-cultivation procedure was studied by FTIR spectroscopy and subsequently compared to commercial chitin as shown in Figure 3(b). The experimentally recovered chitin has a typical *α*-chitin structure with characteristic absorbance bands around 3260.60, 2921.03, 1621.5, 1559, 1516.08, 1403.85, and 1025.6 cm^−1^. In fact, for both spectra, the presence of a peak having a stretching wavelength at 1621.5 cm^−1^ is a characteristic of intramolecular hydrogen bonding CO-HOCH_2_ [5]. The peak having an absorbance at 3260 cm^−1^ indicates the presence of the NH group which reflects the vibratory modes involved in intermolecular hydrogen bonding, while peaks at 2921.03 cm^−1^ reflect symmetrical and asymmetric stretching in the C-H bond. Nevertheless, the absence of absorbance at 1540 cm^−1^ suggests that there is no protein contamination in the recovered chitin.

## 4. Conclusion

In the present research, Taguchi and Box–Behnken designs were employed to optimize the medium composition and the culture conditions for the production of proteases by co-culture of *Aeribacillus pallidus* VP3, *Lysinibacillus fusiformis* C250R, and *Anoxybacillus kamchatkensis* M1V. To the best of our knowledge, this is the first report of a co-culture of three strains used for the fermentation of shrimp biowaste to produce proteases with shrimp powder as a source of carbon/nitrogen and to extract chitin. The obtained results suggest that the biological treatment (co-fermentation) of shrimp by-product powder using three bacterial strains could be considered as an effective pretreatment to produce proteases and a chitin of a high quality.

## Figures and Tables

**Table 1:** Effect of increased shrimp by-product concentration on bacterial growth and protease production by *Anoxybacillus kamchatkensis* M1V.

Concentration of shrimp by-product (g/L)	Protease activity (U/mL)	Biomass (g/L)	Yield (IU protease/g of added by-product)
10	1,200	1.8	120,000
15	1,600	3	106,666
20	1,850	6.07	92,500
25	2,000	7	80,000
30	1,400	5	466,66
35	1,150	5.04	32,857
40	1,000	3	25,000

**Table 2:** Definition of the factors and their corresponding levels studied by Taguchi's L27 design.

Code	Variables	Level 1	Level 2	Level 3
*X*1	Shrimp by-product (g/L)	20	30	40
*X*2	Sucrose (g/L)	10	30	50
*X*3	pH	7	9	11
*X*4	Temperature (°C)	37	40	45
*X*5	Agitation (rpm)	150	180	200
*X*6	Inoculum size of strain VP3 (*A*_600 nm_)	0.1	0.2	0.3
*X*7	Inoculum size of strain M1V (*A*_600 nm_)	0.1	0.2	0.3
*X*8	Inoculum size of strain C250R (*A*_600 nm_)	0.1	0.2	0.3
*X*9	Volume of culture (mL)	25	50	75

**Table 3:** Taguchi's L27 experiment plan with their experimental responses.

Run	*X*_2_	*X*_2_	*X*_3_	*X*_4_	*X*_5_	*X*_6_	*X*_7_	*X*_8_	*X*_9_	Protease activity (U/mL)
1	1	1	1	1	1	1	1	1	1	250
2	1	1	1	1	2	2	2	2	2	30
3	1	1	1	1	3	3	3	3	3	157
4	1	2	2	2	1	1	1	2	2	1,400
5	1	2	2	2	2	2	2	3	3	37
6	1	2	2	2	3	3	3	1	1	7,114
7	1	3	3	3	1	1	1	3	3	2
8	1	3	3	3	2	2	2	1	1	5,546
9	1	3	3	3	3	3	3	2	2	286
10	2	1	2	3	1	2	3	1	2	171
11	2	1	2	3	2	3	1	2	3	49
12	2	1	2	3	3	1	2	3	1	7,019
13	2	2	3	1	1	2	3	2	3	26
14	2	2	3	1	2	3	1	3	1	117
15	2	2	3	1	3	1	2	1	2	104
16	2	3	1	2	1	2	3	3	1	1,637
17	2	3	1	2	2	3	1	1	2	237
18	2	3	1	2	3	1	2	2	3	93
19	3	1	3	2	1	3	2	1	3	0
**20**	**3**	**1**	**3**	**2**	**2**	**1**	**3**	**2**	**1**	**8,182**
21	3	1	3	2	3	2	1	3	2	238
22	3	2	1	3	1	3	2	2	1	286
23	3	2	1	3	2	1	3	3	2	43
24	3	2	1	3	3	2	1	1	3	53
25	3	3	2	1	1	3	2	3	2	20
26	3	3	2	1	2	1	3	1	3	150
27	3	3	2	1	3	2	1	2	1	326

**Table 4:** Analysis of Taguchi's modeled results.

Code	L1	L2	L3	L2-L1	Contribution (%)
*X*1	1647.22	1050.33	1033.11	−596.89	8.94
*X*2	1788.44	1020	922.22	−768.44	12.07
*X*3	322.22	1809.56	1611.56	1487.33	12.53
*X*4	131.11	2104.22	1495.33	1973.11	19.07
*X*5	421.33	1599	1710.33	1177.67	10.34
*X*6	1915.89	896	918.78	−1019.89	14.89
*X*7	296.89	1459.44	1974.33	1162.56	16.19
*X*8	1513.89	1186.78	1030	−327.11	5.98
*X*9	3386.89	281.33	63	−3105.00	47.48

L1: the first level answer. L2: the second level answer. L3: the third level answer. L2-L1: the difference between the second level answer and the first level answer.

**Table 5:** The coded levels of the different factors studied by the Box–Behnken design.

Factor	Level 1	Level 0	Level 1
Temperature (°C)	39	42	45
Inoculum size of strain VP3 (*A*_600 nm_)	0.05	0.1	0.15
Inoculum size of strain M1V (*A*_600 nm_)	0.2	0.3	0.4
Culture volume (mL)	15	25	35

**Table 6:** Screening of factors by the Box–Behnken design.

Code	Temperature (°C)	Inoculum size of strain VP3 (*A*_600 nm_)	Inoculum size of strain M1V (*A*_600 nm_)	Volume (mL)	Protease activity (U/mL)
1	−1	−1	0	0	5,532
2	−1	1	0	0	4,740
3	1	−1	0	0	7,114
4	1	1	0	0	7,637
5	0	0	−1	−1	9,437
6	0	0	−1	1	6,645
7	0	0	1	−1	7,364
8	0	0	1	1	4,400
9	−1	0	0	−1	5,038
10	−1	0	0	1	1,628
11	1	0	0	−1	6,400
12	1	0	0	1	4,503
13	0	−1	−1	0	9,764
14	0	−1	1	0	9,000
15	**0**	**1**	−**1**	**0**	**11,310**
16	0	1	1	0	5,164
17	−1	0	−1	0	6,168
18	−1	0	1	0	3,864
19	1	0	−1	0	6,650
20	1	0	1	0	6,182
21	0	1	0	−1	5,052
22	0	1	0	1	5,134
23	0	1	0	−1	4,322
24	0	1	0	1	5,882
25	0	0	0	0	5,140
26	0	0	0	0	6,472
27	0	0	0	0	6,491

## Data Availability

No data were used to support this study.
